# Everybody's Page

**Published:** 1891-06-27

**Authors:** 


### EVERYBODY'S PAGE.

ACCORDING to Dr. Coltman, who contributes some interesting information in the *Medical Missionary Journal* upon fevers in China, nearly every person in that Celestial Empire suffers at some period or other of his or her life from small-pox. Vaccination appears to be practised, but very carelessly. Sanitary and medical sciences do not seem to be strong points with the otherwise astute heathen.

THE ladies of Tokio set a noble example to their fellow citizens. The Charity Hospital in that city is supported by their annual subscriptions, and to the Hospital is attached a training-school for nurses, the course of study in which extends over two years, and includes anatomy, physiology, bandaging, theoretical and practical nursing, hygiene, and English. Over sixty graduates of the school are stated to be now in the Hospital, and twenty new candidates are admitted every year.

A PLASTER composed of one part of carbonate of lead in two parts of olive oil applied to sprained joints is considered in Holland to be an efficacious remedy. Dr. Duhamel has been trying the effect in Paris on a number of cases, most of which were sprains of the ankle, and the patients were made to walk as soon as the plaster and retaining dressings had been applied. The sceptical may doubt the healing virtue of the plaster and believe that judicious exercise was not without its share in the cure.

A PARISIAN gentleman has written somewhat pathetically to the Secretary of the Metropolitan Public Gardens Association upon the absence of resting places in London. His strictures appear to be more particularly directed against Lincoln's Inn Fields, through which it seems he has to pass day after day carrying heavy weights. Can he be one of the rival "strong men" in disguise? The fact is, there would be a great many more seats in London thoroughfares but for the protests of the inhabitants, who rightly or wrongly believe that the seats when placed in position are too frequently occupied by most undesirable tenants, whose presence makes it impossible for those who value the first laws of cleanliness and health to use these resting places.

As there is very little doubt we are shortly to have the unmixed blessing of free education, or in other words the minority are to have the unalloyed pleasure of paying for the education of the majority, it seems almost futile to kick against the pricks. But there must be many who are not politicians, and may therefore be credited with having some conscientious feelings on the matter, who still protest against taking away from parents the privilege of doing something for the education of their children. It is far too prevalent a fashion with many classes in these days to think lightly of their duties as burdens which they have a perfect right to transfer to the first pair of unresisting shoulders that is handy.

IN one respect at least the Japanese are a very fortunate race. They are stated on good authority to have an almost entire if not entire immunity from scarlet fever. Even Baelz, who affirms that several mild cases of the disease amongst the people as it shows itself in European countries have come under his notice, admits that he has never seen it amongst Japanese mothers and children. Japanese mothers ought to be very happy people.

DR. MEIERHOF, of New York, claims to have devised a very simple hook for the removal of foreign bodies from the ear in cases where the substance is not removable from the external auditory canal by means of irrigation. The instrument consists of a shank in a handle with a bend at the end of the shank at right angles, the little bend which makes the hook being slightly curved laterally, the object of this being that the point of the hook shall not come in contact with the superior wall of the canal.

AMERICANS are occasionally given to scoffing at some of our institutions as antiquated, but theirs, according to their own showing, are not always counsels of perfection. A writer in the *Medical Record* complains that to be a coroner in New York it is not necessary to be a physician, but to be a politician. This evil is in some measure remedied by the fact that each coroner has a deputy, who must be a physician, but not necessarily a good one. It appears much more necessary for him to be a good Tammany man.

THE following interesting letter, supposed to have been received by a doctor from a fellow country physician, is taken from the *Elk Hart Review* (Indiana). It shows an advanced state of knowledge generally: "Dear Dock I hav a pashunt whos phisicol sines shoes that the windpipe has ulcerated of, and his lung hav drop intoo his stumick. He is unabel to swoller and I feer his stumick tube is gon. I hav giv hym evrything without efeckt. His father is welthy, Onerable, and influenshal. What shal I due. Ans. buy returne male. Yours in neede."

A VERY neat, clear little cookery-book, by Charles W. Forward, of practical vegetarian recipes as used in most of the principal vegetarian restaurants in London, reaches us from Messrs. Virtue, and apart from the boon it would be to the vegetarian pure and simple, it is a great help to the man who still clings to his beef and mutton, but who does not object to varying its use occasionally. Lentil cutlets served round a spinach mould are said to be delicious, and for the benefit of the uninitiated we insert the recipe: Take one pint shelled lentils, two tablespoonfuls of rice, one onion, one carrct, one quart water. Boil all the ingredients into a stiff paste, season with pepper, salt, chopped parsley, and powdered marjoram. Turn out on to a board, divide up into cutlet shape with a large knife; cover the bottom of a frying pan with oil, and after rolling the cutlets in bread crumbs make the oil hot and fry them on both sides; serve round spinach or with tomato sauce.

